# Etiology of Diarrhea in Older Children, Adolescents and Adults: A Systematic Review

**DOI:** 10.1371/journal.pntd.0000768

**Published:** 2010-08-03

**Authors:** Christa L. Fischer Walker, David Sack, Robert E. Black

**Affiliations:** Department of International Health, Johns Hopkins Bloomberg School of Public Health, Baltimore, Maryland, United States of America; University of Virginia, United States of America

## Abstract

**Background:**

Diarrhea is an important cause of morbidity and mortality in all regions of the world and among all ages, yet little is known about the fraction of diarrhea episodes and deaths due to each pathogen.

**Methodology/Principal Findings:**

We conducted a systematic literature review to identify all papers reporting the proportion of diarrhea episodes with positive laboratory tests for at least one pathogen in inpatient, outpatient and community settings that met our inclusion and exclusion criteria. We identified a total of 25,701 papers with possible etiology data and after final screening included 22 papers that met all inclusion and exclusion criteria. Enterotoxigenic *Escherichia coli* and *V. cholerae* O1/O139 were the leading causes of hospitalizations. In outpatient settings, *Salmonella* spp., *Shigella* spp., and *E. histolytica* were the most frequently isolated pathogens.

**Conclusions/Significance:**

This is the first systematic review which has considered the relative importance of multiple diarrhea pathogens. The few studies identified suggest that there is a great need for additional prospective studies around the world in these age groups to better understand the burden of disease and the variation by region.

## Author Summary

Diarrhea is an important cause of illness and death around the world and among people of all ages, but unfortunately we often do not know what specific bacterium or virus causes the illness. We conducted a review of the scientific literature with the goal of finding published studies that identified bacteria and viruses among patients with diarrhea in the community and in hospital settings. We initially found nearly 26,000 papers on this topic but narrowed the list to 22 studies that met all of our specific criteria for inclusion in our review. Among patients hospitalized for diarrhea, *E coli* and *Vibrio cholerae* were found in more than 49% of people living in middle income and poor countries. Among patients who sought care from their doctor on an outpatient basis, *Salmonella* spp., *Shigella* spp., and *E. histolytica* were most often found. In our review we focused on the differences in the distribution of pathogens between patients in inpatient vs. outpatient settings because these estimates may best approximate what we would expect to see if the distribution were applied to global estimates of diarrhea deaths vs. uncomplicated illnesses.

## Introduction

Diarrhea is an important cause of morbidity and mortality in all regions of the world and among all ages [1], [2]. For children 5 years of age and older, adolescents, and adults mild to moderate diarrhea can lead to absenteeism from school or work and may require treatment by a health care provider. More severe diarrhea can lead to hospitalization; serious sequelae such as Guillain Barre' syndrome and hemolytic uremic syndrome; and in some cases death [3], [4].

Though most diarrhea episodes are self limiting and dehydration can usually be controlled with oral rehydration therapy, it would be ideal to be able to prevent diarrhea, especially the more severe episodes which have a higher likelihood of progressing to complications or death. Some prevention strategies such as improved water and sanitation and basic hygiene practices are generalizable and thus do not require knowledge of diarrhea etiology, but others such as vaccines would benefit greatly from a comprehensive understanding of the overall burden of pathogen-specific diarrheal disease.

Recent advances have led to the development of an effective rotavirus vaccine which is now recommended for young children as part of the routine immunization schedule [5]. A vaccine for cholera that could be useful in some settings in all ages has been available for several years, and is now recommended by the WHO for persons living in endemic areas [6]. The number of pathogens that are responsible for diarrheal disease goes far beyond rotavirus and *Vibrio cholerae*; however, the fraction of diarrhea episodes and deaths due to each pathogen is unclear, and thus uncertainty may inhibit prioritization of funding for research and disease control programs.

There have been numerous studies conducted in countries around the world to determine the presence of one or more pathogens in diarrheal stools. While isolated studies provide important pieces of information, it is difficult to draw conclusions with regard to the importance of various pathogens without looking at a complete spectrum of agents simultaneously. We conducted a systematic literature review of diarrhea etiology studies to better understand the likely distribution of pathogen-specific diarrhea episodes and deaths in older children, adolescents and adults. To our knowledge this is the first systematic review designed to compile the data from multiple pathogens which might be applied to annual incidence and mortality rates in these age groups.

## Methods

We searched PubMed/Medline, CAB abstracts, System for Information on Grey Literature in Europe (SIGLE), and all World Health Organization (WHO) Regional Databases for studies published from January 1, 1980 through December 31, 2008 using all combinations of the following search and MeSH terms: “diarrhea”, “etiology”, “pathogen”, “incidence”, “mortality”, “cause of death”, and “gastroenteritis”. The objective of the search was to identify all papers reporting the proportion of diarrhea episodes with positive laboratory tests for at least one pathogen in in-patient, out-patient and community settings that met our inclusion and exclusion criteria. We included studies published in all languages and conducted in children ≥5 years, adolescents and adults with at least 12 mo of surveillance (multiples of 12 mo±1 mo for longer studies) to minimize bias due to seasonality of diarrhea pathogens. We excluded studies enrolling *only* patients with clinical signs of dysentery, i.e. blood in the stool, studies conducted in special populations such as travelers, patients hospitalized for other reasons, or only HIV-positive persons and all individual or outbreak case reports. Studies that did not screen for HIV status and/or did not enroll based on HIV status were included. All exclusion criteria were chosen to ensure study populations represented the general population in the study community. In addition, we assessed all laboratory methods for appropriateness and if either incorrect or inadequately described, we excluded the study.

We conducted individual searches in all databases and combined the results to eliminate duplicates using RefWorks Reference Manager [7]. We first reviewed titles for appropriateness and then all abstracts as the first steps of the screening process. For all abstracts with likely applicable data we ascertained the full text article and screened for inclusion and exclusion criteria. All papers meeting our inclusion and exclusion criteria were then abstracted by 2 trained data abstractors into a standardized database. We abstracted information with regard to study population, study setting, diarrhea definition, prevalence of each pathogen, and diarrhea definition required for inclusion in the study. After completing both abstractions we cross checked the data and rectified any differences. We initially included an extensive group of diarrhea pathogens for data abstraction (Table 1), but included only pathogens with available data in the final analyses. For inclusion in the final analyses presented here, we included only studies that adequately described where study participants were recruited from, i.e. inpatient, outpatient, or community settings.

**Table 1:** Pathogens included in initial abstraction.

Enterohaemorrhagic Escherichia coli (EHEC)	*Campylobacter* spp.	*Yersinia* spp.
Enteroinvasive Escherichia coli (EIEC)	*Aeromonas* spp	*Endolimax nana*
Enterotoxigenic Escherichia coli (ETEC)	*Shigella* spp.	*C. difficile*
Enteropathogenic Escherichia coli (EPEC)	*Salmonella* spp.	*Cryptosporidium* spp.
Enteroaggregative Escherichia coli (EAEC)	Giardia spp.	*E. histolytica*
Calicivirus/Norwalk or related agents/Norovirus	*C. perfringens*	*P. shigelloides*

Laboratory methods for each pathogen were reviewed by a laboratory expert (D. S.). Because laboratory techniques for pathogens such as diarrheagenic *E. coli* and *E. histolytica*, have changed since the mid 1990s, we included all studies with standard laboratory procedures for the time of the study [8], [9].

### Analytic Methods

We first calculated the un-weighted mean, median and inter-quartile range for each pathogen for each type of patient population separately. There was only 1 community study [10] so for all analyses we combined this study with the studies conducted in outpatient settings. We then categorized inpatient and outpatient studies based on the number of pathogens reported by the authors in the methods and results sections of each published study: single pathogen studies, those reporting 2–4 pathogens, and those reporting at least 5 pathogens. For each of these categories we calculated the weighted mean for each pathogen. Among inpatient studies we removed the 1 study conducted in a high income setting to enable separate calculations for high vs. low and middle income countries separately. We stratified studies by inpatient or outpatient status and by the number of pathogens identified in each study to present the best possible summary data to approximate the most likely pathogen distributions for diarrhea mortality and all episodes, respectively.

## Results

**Figure 1:**
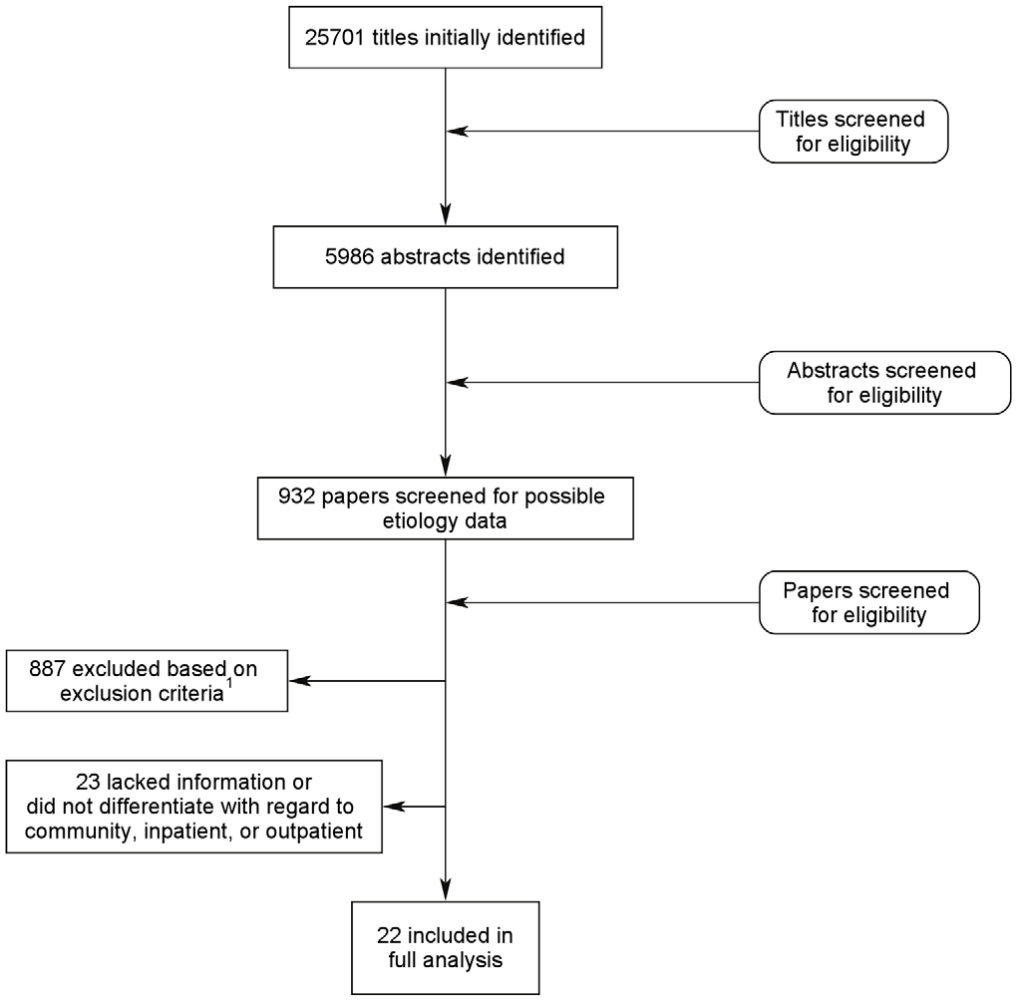
Results of systematic literature review.

We identified 25,701 papers with possible etiology data (Figure 1). After screening 5,986 abstracts and 932 papers, we found 45 that met our inclusion and exclusion criteria. Twenty-two papers met all inclusion and exclusion criteria and described the study populations with regard to inpatient, outpatient or community study populations [10], [11], [12], [13], [14], [15], [16], [17], [18], [19], [20], [21], [22], [23], [24], [25], [26], [27], [28], [29], [30], [31], [32] (Table 2). Twenty three additional papers met initial screening criteria but were subsequently excluded from the analysis presented here because they lacked information with regard to the patient population (i.e. inpatient vs. outpatient) or did not differentiate the results by population type (Table 3) [20], [33], [34], [35], [36], [37], [38], [39], [40], [41], [42], [43], [44], [45], [46], [47], [48], [49], [50], [51], [52], [53].

**Table 2:** Study characteristics of community based, inpatient, and outpatient studies included in the final analyses.

Author (ref)	Country	Date of data collection	Study duration in mos.	Age range in yrs.	Sample size	Pathogens included in study
**Community**						
Spencer [10]	El Salvador	1977	12	≥6	43	ETEC
**Outpatient**						
Hossain [14]	Bangladesh	1975–1984	120	≥5	2488	*Shigella* spp.
Sitbon [17]	Gabon	1980–91	12	≥6	79	Rotavirus
Nath [23]	India	1994–95	24	≥5	916	*Cryptosporidium* spp.
Gassama [12]	Senegal	1997–99	24	≥18	121	EPEC/EAEC, *Salmonella* spp., *Shigella* spp., *Campylobacter* spp., *Aeromonas* spp., *Rotavirus*, *Giardia*, *Cryptosporidium* spp., *E. histolytica*
Lau [15]	China	2001–02	12	≥5	906	Norovirus
MoezArdalan [21]	Iran	2001–02	12	≥5	312	*Shigella* spp.
Al-Gallas [26]	Tunisia	2001–04	36	>18	73	EHEC, EIEC, ETEC, EPEC/EAEC, *Salmonella* spp., *Shigella* spp., *Campylobacter* spp., *Aeromonas* spp., Adenovirus, Rotavirus, Giardia
**Inpatient**						
Oberle [24]	Bangladesh	1975	12	≥5	1459	*Salmonella* spp., *Shigella* spp., *V. cholerae* O1
Zaman [32]	Bangladesh	1978–87	120	≥5	17129	*Shigella* spp.
Black [28]	Bangladesh	1977–79	24	≥10	5171	ETEC, *Salmonella* spp., *Shigella* spp., Rotavirus, Giardia, *E. histolytica*, *V. cholerae* O1, *V. parahaemolytica*
Echeverria [31]	Thailand	1980–81	12	>15	526	ETEC, *Salmonella* spp., *Shigella* spp., Rotavirus, Giardia, *E. histolytica*, *V. cholerae* O1, *V. parahaemolytica*
Watson [19]	UK	1982	12	≥12	515	*Salmonella* spp., *Shigella* spp., *Campylobacter* spp., *Aeromonas* spp., *P. shigelloides*, *C. difficile*
Poocharoen [25]	Thailand	1983–93	12	5–15	17	*Campylobacter* spp.
Baqui [27]	Bangladesh	1983–84	12	≥5	1569	ETEC, *Shigella* spp., *Campylobacter* spp., Giardia, *E. histolytica*, *V. cholerae* O1
Wasfy [18]	Egypt	1986–93	96	5–23	6278	*Salmonella* spp., *Shigella* spp., *Campylobacter* spp.
Brandonisio [29]	Italy	1987–94	84	9–14	28	*Cryptosporidium* spp.
Lim [16]	Singapore	1989–90	12	≥6	5181	*Campylobacter* spp.
Germani [13]	New Caledonia	1990–91	24	≥15	420	ETEC, EPEC/EAEC, *Salmonella* spp., *Shigella* spp., *Campylobacter* spp., Rotavirus, Giardia, *E histolytica*, *C. difficile*
Das [54]	India	1989–91	24	≥5	45	*Cryptosporidium* spp.
Vilchis-Guizar [30]	Mexico	1995	12	39–51	2379	*V. cholerae* O1
Lau [15]	China	2001–2002	12	≥5	240	Norovirus
Nagamani [22]	India	2003–06	48	>5	906	*Cryptosporidium* spp.

**Table 3:** Study characteristics of studies meeting inclusion criteria but excluded from final analysis because the study population (i.e. community-based, inpatient, or outpatient) was not given or results were not stratified by group.

Author (ref)	Country	Date of data collection	Study duration (months)	Age in yrs (mean ± range)	Sample size	Pathogens included in study
Echevarria [55]	Thailand	1982–83	12	≥5	177	ETEC, *Shigella* spp., *Aeromonas* spp., *V. cholerae*, *V. parahaemolyticus*
Chatterjee [41]	India	1982–83	24	5–14	46	EIEC, EPEC, *Salmonella* spp., *Shigella* spp., *Campylobacter* spp., Rotavirus, Giardia, *E. histolytica*, *V. cholerae*, *V. parahaemolyticus*, *P. shigelloides*
Cabrita [39]	Portugal	1984–89	72	≥5	1012	*Salmonella* spp., *Shigella* spp., *Campylobacter* spp.
Rahman [48]	Bangladesh	1985	12	≥6	577	*Cryptosporidium* spp.
Bingnan [38]	Bangladesh	1987–89	24	≥5	2370	Rotavirus
Zvizdic [53]	Bosnia	1988–1991	48	5–7	70	Rotavirus
Cassel-Beraud [40]	Madagascar	1988–89	12	6–14	113	ETEC, *Salmonella* spp., *Shigella* spp., *Campylobacter* spp., Rotavirus, Giardia, *E. histolytica*
Zaman [20]	Saudi Arabia	1989–90	12	≥5	901	*Campylobacter* spp.
Libanorne [47]	Italy	1984–87	24	16–96	1681	Giardia
Katsumata [46]	Indonesia	1992–93	12	≥5	211	*Cryptosporidium* spp.
Samonis [49]	Greece	1992–94	36	≥15	1420	EPEC, *Salmonella* spp., *Shigella* spp., *Campylobacter* spp., *Aeromonas* spp.
Simadibrata [50]	Indonesia	1995–2000	72	(42±14)	207	*Shigella* spp.,Giardia, *E. histolytica*
Akinyemi [34]	Nigeria	1995–96	12	≥6	642	EHEC, EIEC, ETEC, EPEC, *Salmonella* spp., *Shigella* spp., *Aeromonas* spp.
Faruque [42]	Bangladesh	1996–2001	72	≥5	5779	ETEC, EPEC, *Salmonella* spp., *Shigella* spp., *Campylobacter* spp., Rotavirus, *E histolytica*, *V. cholerae*
Bern [37]	Guatemala	1997–98	12	≥5	514	*Cryptosporidium* spp.
Gambhir [44]	India	1997–2000	36	≥15	145	Giardia, *Cryptosporidium* spp., *E. histolytica*
Battikhi [36]	Jordan	1997–2001	48	≥5	560	EPEC, *Salmonella* spp., *Shigella* spp., *Campylobacter* spp., Rotavirus
von Seidlein [52]	Multi-site Asia^1^	2002	12	>5	8253	*Shigella* spp.
Abreu-Acosta [33]	Spain	2002–2004	24	≥13	17	*Cryptosporidium* spp.
Hamedi [45]	Iran	2003	12	5–7	31	*Cryptosporidium* spp.
Uchida [51]	Nepal	2003–04	12	≥5	645	Rotavirus
Amarilla [35]	Paraguay	2004–05	24	18–95	801	Rotavirus
Feizabadi [43]	Iran	2004–05	12	5–14	79	*Campylobacter* spp.

1 China, Thailand, Indonesia, Vietnam, Pakistan, Bangladesh.

In table 4 we present the unweighted mean and median proportion of stools which tested positive for each pathogen in both in- and out-patient settings. In this analysis *V. cholerae* O1/O139 and ETEC were the leading causes of hospitalization. In inpatient populations *Aeromonas* spp. *Yersinia* spp. *Cryptosporidium* spp., *V. parahaemolyticus*, *P. shigelloides*, and *C. difficile* were each found in <2% of patients. In out-patient settings, *Salmonella* spp., *Shigella* spp., and *E. hystolitica* were isolated the most frequently. In outpatient populations, EHEC, *Campylobacter* spp., *Aeromonas* spp. and *Yersinia* spp. were found in <2% of patients. Very few studies tabulated data such that the co-occurance of more than one pathogen in a diarrheal stool could be ascertained and few tested a broad enough spectrum of pathogens to be able to quantify the proportion of episodes from which no currently recognized pathogen could be identified.

**Table 4:** Isolation of pathogens by inpatient and outpatient/community settings.

	Inpatient (15 total studies)		Outpatient/Community (8 total studies)	
Pathogen	Mean (# of studies included)	Median [IQR]	Mean (# of studies included)	Median [IQR]
Adenovirus	–	–	7 (1)	7 [NA]
*Aeromonas* spp.	0.2 (1)	0.2 [NA]	0.8 (2)	0.8 [0.4, 1.2]
*Campylobacter* spp.	4.1 (6)	1.4 [0.5, 5.9]	1.5 (2)	1.5 [1.5, 1.6]
*C. difficile*	1.5 (2)	1.5 [0.7, 2.2]	–	–
*Cryptosporidia* spp.	1.9 (3)	1.3 [0.9, 2.2]	6.4 (2)	6.9 [4.9, 7.0]
*E. histolytica*	3.1 (3)	2.3 [2.1, 3.3]	10.7 (1)	10.7 [NA]
EPEC/EAEC	4.0 (1)	4.0 [NA]	4.7 (2)	4.7 [3.7, 5.6]
ETEC	14.0 (4)	9.5 [3.4, 20.2]	5.9 (2)	5.7 [3.5, 8.1]
EHEC	–	–	0 (1)	0 [NA]
EIEC	–	–	4 (2)	4 [2.7, 5.3]
*Giardia* spp.	2.5 (3)	2.2 [2.2, 2.6]	2.5 (2)	2.5 [1.2, 3.7]
Norovirus	10 (1)	10 [NA]	8.5 (1)	8.5 [NA]
*P. shigelloides*	0.2 (1)	0.2 [NA]	–	–
Rotavirus	3.1 (3)	4.1 [2.0, 4.7]	2.1 (3)	2.3 [1.9, 2.4]
*Salmonella* spp.	8.4 (5)	3.3 [2.3, 11.4]	20.4 (2)	20.4 [13.5, 27.3]
*Shigella* spp.	6.5 (8)	4.3 [3.0, 10.2]	19.6 (4)	18.5 [10.3, 27.8]
*V. cholerae* (O1/O139)	15.3 (5)	14.0 [11.9, 30.2]	–	–
*V. parahaemolyticus*	1.6 (2)	1.6 [0.8, 2.4]	–	–
*Yersinia* spp.	0 (1)	0 [NA]	0 (1)	0 [NA]

In Table 5 we present the analysis of inpatient studies stratified by the number of pathogens sought among those studies conducted in low and middle income countries. We separately present the results for the single analysis which included more than 4 pathogens conducted in a high income setting [19]. There were very few single pathogen studies thus it is difficult to identify a trend as one progresses from single to comprehensive studies with at least 5 pathogens. In the studies conducted in low and middle income countries which identified at least 5 pathogens, 28.1% of hospitalized patients had tested positive for ETEC and 20.7% tested positive for *V. cholera* O1/O139. For high income/low mortality countries, one study found that 14% of hospitalized patients tested positive for *Campylobacter* spp. followed by 11.5% of samples testing positive for *Salmonella* spp. [19].

**Table 5:** Isolation of single vs. multiple pathogens among inpatients.

	Weighted mean % (# of studies) for low and middle income countries			One study representing high income countries [19]
Pathogen	Single Pathogen Studies	2–4 Pathogens Studies	>4 Pathogen Studies	Percent of patients positive for each pathogen
EPEC/EAEC	–	–	4 (1)	
ETEC	–	–	28.2 (4)	
*Salmonella* spp.	–	2.7 (2)	12.3 (2)	11.5
*Shigella* spp.	0.2 (1)	3.7 (2)	6.7 (4)	3.1
*Campylobacter* spp.	0.5 (2)	2.3 (2)	5.7 (2)	14
*Cryptosporidia* spp.	1.3 (3)	–	–	
*Aeromonas* spp.	–	–		0.2
*Yersinia* spp.	–	–	0 (1)	
*Giardia* spp.	–	–	2.2 (3)	
Rotavirus	–	–	3.9 (3)	
Norovirus	10 (1)	–	–	
*V. cholerae* O1/O139	11.9 (1)	30.2 (1)	20.7 (3)	
*V. parahaemolyticus*	–	–	0.3 (2)	
*E. histolytica*	–	–	3.8 (3)	
*P. shigelloides*	–	–		0.2
*C. difficile*	–	–	0.2 (1)	2.9

For outpatient studies we only identified studies of single pathogens and those which looked for more than 4 pathogens (Table 6). The difference in proportion of stools testing positive for a particular pathogen is most noticeable for *Shigella* spp. where 34.3% of episodes were positive for *Shigella* in studies that sought only that pathogen, vs. only 9.4% positive among studies which looked for 5 of more pathogens.

**Table 6:** Isolation of single vs. multiple pathogens for outpatient studies.

	Weighted Mean (# of studies)	
Pathogen	Single Pathogen	>4 Pathogens
EPEC/EAEC	–	5.2 (2)
ETEC	4.7 (1)	4.6 (1)
EHEC	–	0 (1)
EIEC	–	2.6 (2)
*Salmonella* spp.	–	17 (2)
*Shigella* spp.	35.4 (2)	9.3 (2)
*Campylobacter* spp.	–	1.5 (2)
*Cryptosporidia* spp.	6.9 (1)	2 (1)
*Aeromonas* spp.	–	1 (2)
*Yersinia* spp.	–	0(1)
*Giardia* spp.	–	3.1 (2)
*E. histolytica*	–	10.7 (1)
Rotavirus	2.3 (1)	2.1 (2)
Norovirus	8.5 (1)	–
Adenovirus	–	2.6 (1)

## Discussion

This is the first systematic review which has considered the relative importance of multiple diarrhea pathogens for all regions of the world among children 5 years and older, adolescents, and adults using studies published in the peer reviewed literature. We stratified our results by inpatient vs. outpatient settings because it is likely that the distribution of pathogens differs by diarrhea severity. We found ETEC and *V. cholerae* O1/O139 to be the most frequently isolated pathogens among patients hospitalized for diarrhea; together they were observed in more than 49% of samples from patients in low and middle income countries. Because these studies were conducted in cholera endemic areas this is not surprising; the importance of cholera will depend on whether the study was done in an endemic or epidemic area thus these results are not possible to generalize to all countries. Rotavirus, which is known to be a leading cause of death among young children, was not found to be as important among older persons providing additional evidence suggesting immunity with increasing age.

In outpatient settings, *Salmonella* spp., *Shigella* spp., and *E. histolytica* were the most frequently isolated pathogens. Because little is known about the care-seeking behavior for community-acquired diarrhea among children 5 years of age and older and adults, additional data are needed in this age group to determine the distribution of pathogens in the community. Because blood in the stool is common for illnesses due to *Shigella* spp., Campylobacter spp., and *E. histolytica* and may occur with *Salmonella* spp. it is possible that the isolation of these pathogens would be higher than in a true community-based setting due to an increase in care-seeking behavior for illnesses with the presence of blood in the stool. We only identified one community-based study; thus, separate estimates for outpatient and community studies were not possible.

The overall scarcity of the data used to produce these estimates is a major limitation. This is particularly concerning when generalizing across regions and when making assumptions about variations which are likely among low, middle, and high income countries based on variation in geography and risk factors. Given the few studies meeting our criteria for inclusion in the review, it is not possible to account for the additional differences in study populations by region or over time which might have also influenced the spectrum of pathogens due to changes in pathogens chosen for isolation, pathogens circulating in a community, and baseline characteristics of the study population.

An additional limitation of this review is the time span of the included studies and thus heterogeneity of laboratory methods for some key pathogens. In the last 30 years, diagnostic methods have evolved for many pathogens, such as diarrheagenic *E. coli* and *E. histolytica*. New laboratory methods, including PCR, and antigen detection assays have increased sensitivity and decreased risk of misclassification substantially. Because some reports included in this review used older laboratory methods there is a risk that data from these may under- or over-represent the prevalence of selected pathogens. However, because of the overall paucity of data we chose to include these studies however caution should be taken when interpreting the results for these selected pathogens for which laboratory methods have improved dramatically over the past 30 years.

In this review we stratified studies by those that sought a single pathogen and those that considered multiple pathogens. Because single pathogen studies often pick study sites based on a known prevalence of a particular pathogen it can be expected that the observed rates would be higher than in studies where multiple pathogens are being isolated. This was especially true for *Shigella* spp. where we found the weighted mean dropped from 35.3% in the single pathogen studies to 9.4% in the multiple pathogen studies. In addition, outpatient studies did not look specifically for some pathogens such as *V. cholerae* O1/O139 thus limiting the inference about non severe episodes.

We recognize that we did not capture the true burden of every possible pathogen that might cause diarrhea because many pathogens occur in outbreaks and these may not have been included in these ongoing disease surveillance studies. For example, we only found one study that included detection of norovirus [15] meeting our study inclusion criteria of at least 12 mo of surveillance. Norovirus is known to be seasonal and a frequent cause of epidemics, so may be underestimated in our review. Similarly because we did not include outbreak data, pathogens that are more typically observed in outbreaks may have been missed if they were not known to be endemic in the study area.

Because we identified very few studies that tested for 5 pathogens or more and most were from South Asia, we were not able to assess regional differences in pathogen importance. For pathogens that are not known to be prevalent globally such as *V. cholerae* O1/O139 this is especially problematic. Ideally unique pathogen distributions would be developed for each region and for large countries, such as Brazil, China, and India. National level community-based surveillance and inpatient reports would enable countries to better understand the local burden of disease by pathogen and better design prevention programs.

In this analysis we have treated all data as isolated proportions yet we recognize that this is not the case for many diarrhea episodes. Many patients have multiple pathogens and likewise for some patients, no pathogen is found. Because we had very few studies seeking multiple pathogens and even fewer reporting mixed infections, we were not able to conduct a more complex analysis to control for the role of multiple infections. We also recognize that the identification of a pathogen in the stool does not necessary mean that it is the cause of the illness. Many patients are asymptomatic carriers and thus the prevalence of some pathogens might be found at the similar proportions in healthy individuals. These pathogens have a lower pathogenicity than those that are never or rarely identified in the stools of asymptomatic individuals. Only one study in our final data set provided data for asymptomatic controls, thus a full analysis to control for asymptomatic carriage was not possible.

This study is the first to systematically review the literature on the etiology of diarrhea in children ≥5 years of age, adolescents and adults and provides an important overview of the distribution of pathogens responsible for both infection and possible death. The few studies identified suggest that great caution must be taken when interpreting these limited data. Many limitations have been identified suggesting the need for additional prospective studies around the world in these age groups. Understanding the burden of pathogen specific diarrheal disease and the variation by region is important for planning effective control programs for the overall reduction of diarrhea disease among persons of all ages.

## Supporting Information

Checklist S1 PRISMA checklist.
(0.07 MB DOC)
